# Skeletal Muscle Texture Assessment Using Ultrasonography: Comparison with Magnetic Resonance Imaging in Chronic Kidney Disease

**DOI:** 10.1177/01617346241255879

**Published:** 2024-05-28

**Authors:** Thomas J. Wilkinson, Luke A. Baker, Emma L. Watson, Katerina Nikopoulou, Christina Karatzaferi, Matthew PM. Graham-Brown, Alice C. Smith, Giorgos K. Sakkas

**Affiliations:** 1 Leicester Biomedical Research Centre (BRC), Leicester Diabetes Centre, University of Leicester, Leicester, UK; 2 Leicester Kidney Lifestyle Team, Department of Population Health Sciences, University of Leicester, Leicester, UK; 3 Department of Respiratory Sciences, University of Leicester, Leicester, UK; 4 Department of Cardiovascular Sciences, University of Leicester, Leicester, UK; 5 Department of Physical Education and Sport Sciences, University of Thessaly, Trikala, Greece

**Keywords:** muscle texture, ultrasound, ultrasonography, echo intensity, magnetic resonance imaging, chronic kidney disease

## Abstract

Skeletal muscle dysfunction is common in chronic kidney disease (CKD). Of interest is the concept of “muscle quality,” of which measures include ultrasound-derived echo intensity (EI). Alternative parameters of muscle texture, for example, gray level of co-occurrence matrix (GCLM), are available and may circumvent limitations in EI. The validity of EI is limited in humans, particularly in chronic diseases. This study aimed to investigate the associations between ultrasound-derived parameters of muscle texture with MRI. Images of the thigh were acquired using a 3 Tesla MRI scanner. Quantification of muscle (contractile), fat (non-contractile), and miscellaneous (connective tissue, fascia) components were estimated. Anatomical rectus femoris cross-sectional area was measured using B-mode 2D ultrasonography. To assess muscle texture, first (i.e., EI)- and second (i.e., GLCM)-order statistical analyses were performed. Fourteen participants with CKD were included (age: 58.0 ± 11.9 years, 50% male, eGFR: 27.0 ± 7.4 ml/min/1.73m^2^, 55% Stage 4). Higher EI was associated with lower muscle % (quadriceps: β = −.568, *p* = .034; hamstrings: β = −.644, *p* = .010). Higher EI was associated with a higher fat % in the hamstrings (β = −.626, *p* = .017). A higher angular second moment from GLCM analysis was associated with greater muscle % (β = .570, *p* = .033) and lower fat % (β = −.534, *p* = .049). A higher inverse difference moment was associated with greater muscle % (β = .610, *p* = .021 and lower fat % (β = −.599, *p* = .024). This is the first study to investigate the associations between ultrasound-derived parameters of muscle texture with MRI. Our preliminary findings suggest ultrasound-derived texture analysis provides a novel indicator of reduced skeletal muscle % and thus increased intramuscular fat.

## Introduction

Skeletal muscle dysfunction and sarcopenia are common in people with chronic kidney disease (CKD),^1^ reducing physical function and quality of life, and increasing the risk of frailty and mortality.^2^ Increasing attention is being paid to the assessment of skeletal muscle in CKD. Simple, portable, and cost-effective methodologies, such as ultrasound, now offer an accurate bedside means to assess skeletal muscle and of particular interest is the concept of “muscle quality,” qualities beyond mass including histological, imaging, metabolic, and functional/impairment assessments.^3,4^ Common measures of muscle quality include echo intensity (EI), a measure of muscle texture, and a metric that measures grayscale pixels’ contrast, and consistency. EI is believed to be a parameter of fatty infiltration of the muscle^5^ and studies using magnetic resonance imaging (MRI) have suggested that EI may be an indication of intramuscular adiposity.^6-9^ Whilst fibrous tissue may also influence ultrasound-derived EI,^10^ evidence for this is scarce and derived from only two studies: one is an animal study and another based on the autopsy of a 62-year-old woman with amyotrophic lateral sclerosis.^10,11^

Alongside EI, alternative parameters of muscle texture, such as gray level of co-occurrence matrix (GCLM) (a texture analysis approach accounting for spatial distribution of the pixels) are available and may in fact circumvent methodological limitations in EI.^12,13^ Data concerning the validity of EI versus MRI is limited in humans,^8^ particularly in chronic disease states, and no research has explored the association between GCLM and MRI-derived muscle composition markers. While additional validity studies in humans utilizing MRI are needed to help understand what EI represents,^11^ the aim of this preliminary analysis was to investigate the associations between ultrasound-derived parameters of muscle texture with MRI in those living with chronic kidney disease.

## Materials and Methods

### Participants

This is an exploratory secondary analysis of data taken from the ExTra-CKD trial (ISRCTN36489137) (14). In brief, patients with CKD stages 3b–5 were recruited from nephrology outpatient clinics in Leicester, UK. Exclusion criteria included being aged <18 years and physical impairment preventing undertaking exercise. All patients gave written informed consent, and the trial was conducted in accordance with the Declaration of Helsinki. The study was given favorable ethical opinion by the National Research Ethics Committee (no. 13/EM/0344).

### MRI Acquisition

**Figure 1.**
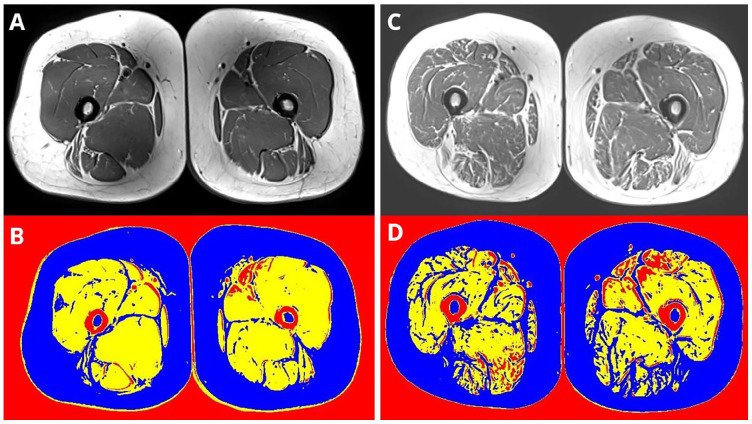
Quantification of muscle, fat, and miscellaneous components of the muscle taken from MRI Figure shows image from original MRI (top row) and then quantification of muscle and fat (bottom row). Figure shows two male study participants with similar thigh area and represents relatively preserved muscle composition (left, images A and B, male, aged 58 years, eGFR 28 ml/min/1.73 m^2^, and poor muscle composition (right, images C and D, male, aged 50 years, eGFR 32 ml/min/1.73 m^2^). Yellow = muscle (contractile), Blue = fat (non-contractile), Red = miscellaneous (connective tissue, fascia) tissue.

Images of the thigh (including quadriceps and hamstrings) were acquired from the participants’ right leg using a 3 Tesla MRI scanner (Siemens Skyra). Images (from proximal border of the patella to the superior aspect of the femur) were obtained in the axial plane using a T1 turbo spin-echo sequence (slice thickness, 5 mm with no gap between slices; repetition time/echo time, 873 ms/14 ms; field of view, 450 × 309.4 mm; in-plane resolution, 0.879 × 0.879 mm).^14^ Analysis of the MRI slices was performed in a customized software program as previously described in those receiving dialysis.^15–19^ Based on variations in signal intensity, this allowed the quantification of muscle (contractile) and fat (non-contractile) components of the total cross-sectional area (CSA) of the muscle compartment of the leg (excluding subcutaneous adipose tissue (SAT) and bones) (Figure 1). A very low signal intensity, below that of the muscle threshold, was assigned as miscellaneous (connective tissue, fascia). The slices with the largest cross-sectional area in the thigh were analyzed. Each slice was measured three times and the average value was taken. The following variables were measured in the quadriceps and hamstring muscles: total CSA, muscle CSA and %, fat CSA and %, miscellaneous CSA and %, and thigh SAT. Consistent with studies using the same technique,^16^ the intra-subject coefficient of variation was between 0% and 5% for all variables.

### Ultrasound Acquisition

Anatomical rectus femoris CSA (RF-CSA) was measured from the right leg using B-mode 2D ultrasonography (Hitachi EUB-6500; probe frequency, 7.5 MHz) under resting conditions. As previously described,^12,14,20^ imaging was performed at the midpoint between the greater trochanter and the superior aspect of the patella on the mid-sagittal plane of the thigh. The probe was placed transversally to the longitudinal axis of the thigh forming a 90° angle to the skin surface. RF-CSA was calculated as an average of three consecutive measurements with <10% variation. A single researcher performed all scans with an interclass correlation coefficient of 0.95. RF-CSA is a reliable index of MRI-derived total quadriceps volume^14^ and an indicator of sarcopenia in CKD.^20^

Analysis was performed using ImageJ 1.52v (National Institutes of Health, USA) by a single observer. To assess muscle texture, first (i.e., EI)- and second (i.e., GLCM)-order statistical analyses were performed. EI was determined using the distribution of the in-built histogram tool (0: black and 255: white). GLCM was assessed using a bespoke plugin macro (GLCM Texture v0.4, Julio Cabera). As previously described,^12^ five GLCM features were quantified: angular second moment (ASM), entropy, or inverse difference moment (IDM), correlation, and contrast.

### Statistical Analysis

Data is shown for the 14 participants with an MRI scan at baseline and GLCM data available. The association of markers of muscle texture were compared with MRI-derived markers of muscle composition using univariate general linear modeling adjusted for age, sex, and eGFR. Analysis was conducted using IBM SPSS Statistics (version 29.0.1.0).

## Results

**Figure 2.**
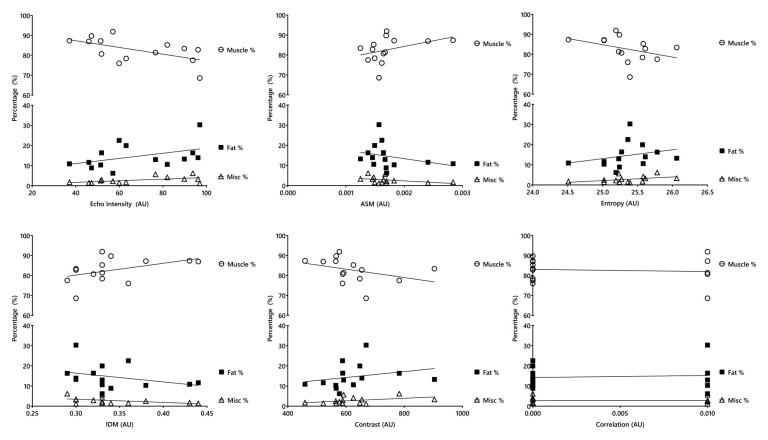
Association between ultrasound-derived texture analysis and quadriceps MRI-derived muscle composition variables. AU = arbitrary unit; ASM = angular second moment (energy); IDM = inverse difference moment (homogeneity).

**Figure 3.**
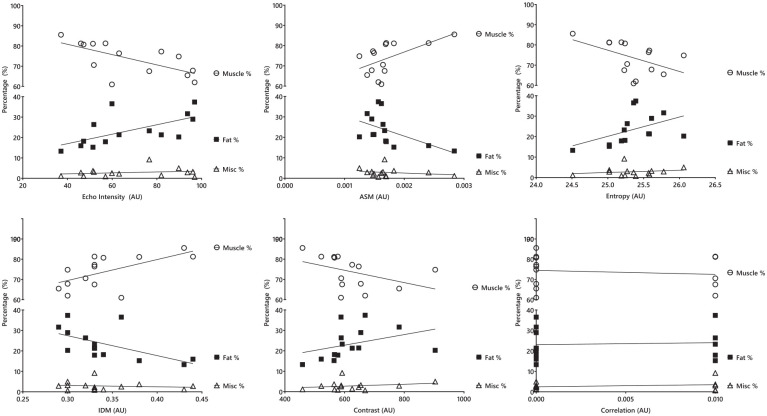
Association between ultrasound-derived texture analysis and hamstrings MRI-derived muscle composition variables. AU = arbitrary unit; ASM = angular second moment (energy); IDM = inverse difference moment (homogeneity).

The mean age was 58.0 ± 11.9 years, half were male, and half were of White British ethnicity. Mean eGFR was 27.0 ± 7.4 ml/min/1.73 m^2^ (Table 1). The association between ultrasound-derived texture analysis and MRI-derived muscle composition variables is shown in Table 2 (visually shown in Figures 2 and 3). In the quadriceps and hamstrings, a higher EI was significantly associated with lower muscle % (quadriceps: β = −.568, *p* = .034; hamstrings: β = −.644, *p* = .010). Higher EI was associated with a higher fat % in the hamstrings (β = −.626, *p* = .017). In the hamstrings, a higher ASM was associated with greater muscle % (β = .570, *p* = .033) and lower fat % (β = −.534, *p* = .049). Similarly, a higher IDM was also associated with greater muscle % (β = .610, *p* = .021 and lower fat % (β = −.599, *p* = .024). Greater thigh SAT was associated with lower correlation values (β = −.594, *p* = .025). No associations were seen between MRI-derived muscle composition variables and entropy or contrast. No variables were associated with miscellaneous markers.

**Table 1.** Participants’ Basic Characteristics.

	All participants (*N* = 14)
Age (years)	58.0 (±11.9)
Sex (male *n*, %)	7 (50%)
Ethnicity (*n*, %)^¥^	
White British	7 (50%)
South Asian	4 (29%)
eGFR (ml/min/1.73 m^2^)^¥^	27.0 (±7.4)
CKD stages	
Stage 3b	5 (45%)
Stage 4	6 (55%)
Hypertension (*n*, %)^¥^	6 (43%)
Diabetes (*n*, %)^¥^	1 (7%)
BMI (kg/m^2^)	29.0 (±4.0)
Quadriceps composition	
Muscle %	82.6 (±6.2)
Fat %	14.6 (±6.3)
Misc. %	2.8 (±1.6)
Hamstrings composition	
Muscle %	73.8 (±8.0)
Fat %	23.4 (±7.7)
Misc. %	2.8 (±2.2)
Thigh SAT (cm^2^)	133.6 (±56.6)

Unless otherwise stated, data are shown as mean and standard deviation.

¥ Missing data: ethnicity, *n* = 3; co-morbidities, *n* = 4; eGFR, *n* = 3.

**Table 2.** Association Between Ultrasound-Derived Texture Analysis and MRI-Derived Muscle Composition Variables.

	Quadriceps						Hamstrings						Thigh SAT	
	Muscle %		Fat %		Misc. %		Muscle %		Fat %		Misc. %			
	β	*p*	β	*p*	β	*p*	β	*p*	β	*p*	β	*p*	β	*p*
EI	**–.568**	.034*	.427	.128	.522	.056	**–.664**	.010*	**.626**	.017*	.217	.457	.148	.614
ASM	.387	.172	–.280	.331	–.398	.159	**.570**	.033*	**–.534**	.049*	–.196	.501	–.289	.316
Entropy	–.379	.181	.267	.356	.416	.139	–.495	.072	.461	.097	.180	.539	.490	.075
IDM	.456	.102	–.337	.238	–.446	.110	**.610**	.021*	**–.599**	.024*	–.396	.699	–.118	.688
Correlation	–.094	.748	.093	.751	.020	.946	–.133	.650	.100	.734	.137	.641	**–.594**	.025*
Contrast	–.374	.187	.257	.375	.435	.120	–.419	.135	.368	.195	.231	.428	.475	.086

Data shown as standardized beta. Statistical significance recognized as *p* < .005, indicated by * and shown in bold.

EI = echo intensity; ASM = angular second moment (energy); IDM = inverse difference moment (homogeneity); SAT = subcutaneous adipose tissue.

## Discussion

This is the first study to preliminary investigate the associations between ultrasound-derived parameters of muscle texture with MRI in people living with CKD. In a small sample, our findings show that ultrasound-derived EI may be a useful marker of reduced skeletal muscle %, and thus increased intramuscular fat %. EI did not show any association with miscellaneous components that is, connective tissue, fascia. These findings support the previously held notion that EI may be a marker of intramuscular adiposity.^8,9^

In the context of the known limitations of the EI between scanners,^11^ we found GLCM-markers ASM and IDM may be alternative markers of muscle composition. A key advantage of GLCM is that its parameters are robust to changes in ultrasound scanner settings (e.g., gain, frequency)^13^ as it takes into account the two-dimensional distribution of pixels. We found higher ASM and IDM were associated with greater muscle % and lower intramuscular fat %. Overall, these texture markers represent greater image homogeneity, and likely indicate lower muscular infiltration of fat.

Our study is strengthened by an MRI assessment coupled with a comprehensive panel of markers from ultrasound-derived texture analysis. Overall, studies exploring the relationship between ultrasound and MRI remain small in number, and further validation work is needed. As an exploratory secondary analysis, our sample is limited in its size and the analysis may not have been powered to detect significant differences across variables. Another issue that needs to be acknowledged is that some MRI images appeared with susceptibility artifacts at the edge of the image due to the high field of view (FOV). Those artifacts could affect image analysis, however, the appropriate correction factors have been applied for normalizing the color intensity (B0 correction) before analysis. Further, collaborative efforts focused on methodology are needed to improve the consistency and quality of the literature.^11^

Our findings have important practical applications for the care of people with CKD. Higher muscle fat infiltration is an indication of lower muscle quality, and lower muscle quality is associated with adverse clinical and patient outcomes such as lower physical performance and quality of life.^4^ Identifying those with low muscle quality may help stratify those at risk and help healthcare professionals implement early interventions, such as exercise, that may have favorable implications on muscle physiology.

## Conclusion

In summary, simple ultrasound-derived texture analysis may provide a novel indicator of intramuscular fat in people with CKD and have the potential to become a measure of muscle health and intervention targets for sarcopenia.

## References

[1] Duarte MP Almeida LS Neri SGR Oliveira JS Wilkinson TJ Ribeiro HS , et al Prevalence of sarcopenia in patients with chronic kidney disease: a global systematic review and meta-analysis. J Cachexia Sarcopenia Muscle. 2024;15(2):501-12. 10.1002/jcsm.13425 PMC10995263 38263952

[2] Wilkinson TJ Miksza J Yates T Lightfoot CJ Baker LA Watson EL , et al Association of sarcopenia with mortality and end-stage renal disease in those with chronic kidney disease: a UK Biobank study. J Cachexia Sarcopenia Muscle. 2021;12(3):586-98. 10.1002/jcsm.12705 PMC8200422 33949807

[3] Cawthon PM Visser M Arai H Ávila-Funes JA Barazzoni R Bhasin S , et al Defining terms commonly used in sarcopenia research: a glossary proposed by the Global Leadership in Sarcopenia (GLIS) Steering Committee. Eur Geriatr Med. 2022;13(6):1239-44. 10.1007/s41999-022-00706-5 PMC9722886 36445639

[4] Avesani CM de Abreu AM Ribeiro HS Brismar TB Stenvinkel P Sabatino A , et al Muscle fat infiltration in chronic kidney disease: a marker related to muscle quality, muscle strength and sarcopenia. J Nephrol. 2023;36(3):895-910. 36719556 10.1007/s40620-022-01553-0 PMC10090035

[5] Perkisas S Baudry S Bauer J Beckwée D De Cock A-M Hobbelen H , et al Application of ultrasound for muscle assessment in sarcopenia: towards standardized measurements. Eur Geriatr Med. 2018;9(6):739-57. 10.1007/s41999-018-0104-9 34674473

[6] Goodpaster BH Stenger VA Boada F McKolanis T Davis D Ross R , et al Skeletal muscle lipid concentration quantified by magnetic resonance imaging. Am J Clin Nutr. 2004;79(5):748-54. 10.1093/ajcn/79.5.748 15113711

[7] Akima H Hioki M Yoshiko A Koike T Sakakibara H Takahashi H , et al Intramuscular adipose tissue determined by T1-weighted MRI at 3T primarily reflects extramyocellular lipids. Magn Reson Imaging. 2016;34(4):397-403. 26743430 10.1016/j.mri.2015.12.038

[8] Young HJ Jenkins NT Zhao Q Mccully KK. Measurement of intramuscular fat by muscle echo intensity. Muscle Nerve. 2015;52(6):963-71. 10.1002/mus.24656 PMC4575231 25787260

[9] Lortie J Rush B Osterbauer K Colgan TJ Tamada D Garlapati S , et al Corrigendum: myosteatosis as a shared biomarker for sarcopenia and cachexia using MRI and ultrasound. Front Rehabil Sci. 2022;3:982949. 36191164 10.3389/fresc.2022.982949 PMC9397885

[10] Pillen S Tak RO Zwarts MJ Lammens MM Verrijp KN Arts IM , et al Skeletal muscle ultrasound: correlation between fibrous tissue and echo intensity. Ultrasound Med Biol. 2009;35(3):443-6. 10.1016/j.ultrasmedbio.2008.09.016 19081667

[11] Stock MS Thompson BJ. Echo intensity as an indicator of skeletal muscle quality: applications, methodology, and future directions. Eur J Appl Physiol. 2021;121(2):369-80. 10.1007/s00421-020-04556-6 33221942

[12] Wilkinson TJ Ashman J Baker LA Watson EL Smith AC. Quantitative muscle ultrasonography using 2D textural analysis: a novel approach to assess skeletal muscle structure and quality in chronic kidney disease. Ultrason Imaging. 2021;43(3):139-48. 10.1177/01617346211009788 PMC8114433 33853450

[13] Paris MT Bell KE Avrutin E Mourtzakis M. Ultrasound image resolution influences analysis of skeletal muscle composition. Clin Physiol Funct Imaging. 2020;40(4):277-83. 10.1111/cpf.12636 32342635

[14] Gould DW Watson EL Wilkinson TJ Wormleighton J Xenophontos S Viana JL , et al Ultrasound assessment of muscle mass in response to exercise training in chronic kidney disease: a comparison with MRI. J Cachexia Sarcopenia Muscle. 2019;10(4):748-55. 10.1002/jcsm.12429 PMC6711420 31054219

[15] Sakkas GK Kent-Braun JA Doyle JW Shubert T Gordon P Johansen KL. Effect of diabetes mellitus on muscle size and strength in patients receiving dialysis therapy. Am J Kidney Dis. 2006;47(5):862-9. 10.1053/j.ajkd.2006.01.013 16632026

[16] Johansen KL Shubert T Doyle J Soher B Sakkas GK Kent-Braun JA. Muscle atrophy in patients receiving hemodialysis: effects on muscle strength, muscle quality, and physical function. Kidney Int. 2003;63(1):291-7. 10.1046/j.1523-1755.2003.00704.x 12472795

[17] Johansen KL Painter PL Sakkas GK Gordon P Doyle J Shubert T. Effects of resistance exercise training and nandrolone decanoate on body composition and muscle function among patients who receive hemodialysis: a randomized, controlled trial. J Am Soc Nephrol. 2006;17(8):2307-14. 10.1681/ASN.2006010034 16825332

[18] Giannaki CD Sakkas GK Karatzaferi C Hadjigeorgiou GM Lavdas E Liakopoulos V , et al Evidence of increased muscle atrophy and impaired quality of life parameters in patients with uremic restless legs syndrome. PLoS One. 2011;6(10):e25180. 10.1371/journal.pone.0025180 PMC3184961 21984901

[19] Sakkas GK Gourgoulianis KI Karatzaferi C Liakopoulos V Maridaki MD Pastaka C , et al Haemodialysis patients with sleep apnoea syndrome experience increased central adiposity and altered muscular composition and functionality. Nephrol Dial Transplant. 2008;23(1):336-44. 10.1093/ndt/gfm559 17890750

[20] Wilkinson TJ Gore EF Vadaszy N Nixon DGD Watson EL Smith AC. Utility of ultrasound as a valid and accurate diagnostic tool for sarcopenia: sex-specific cutoff values in chronic kidney disease. J Ultrasound Med. 2021;40(3):457-67. 10.1002/jum.15421 32780522

